# Rapid Enrichment and Isolation of Polyphosphate-Accumulating Organisms Through 4’6-Diamidino-2-Phenylindole (DAPI) Staining With Fluorescence-Activated Cell Sorting (FACS)

**DOI:** 10.3389/fmicb.2020.00793

**Published:** 2020-04-30

**Authors:** Mia Terashima, Yoichi Kamagata, Souichiro Kato

**Affiliations:** ^1^Bioproduction Research Institute, National Institute of Advanced Industrial Science and Technology (AIST), Sapporo, Japan; ^2^Division of Applied Bioscience, Graduate School of Agriculture, Hokkaido University, Sapporo, Japan; ^3^Bioproduction Research Institute, National Institute of Advanced Industrial Science and Technology (AIST), Tsukuba, Japan

**Keywords:** Polyphosphate-accumulating organisms, flow cytometry, wastewater sludge, bacteria isolation, DAPI

## Abstract

Screening for bacteria with abilities to accumulate valuable intracellular compounds from an environmental community is difficult and requires strategic methods. Combining the experimental procedure for phenotyping living cells in a microbial community with the cell recovery necessary for further cultivation will allow for an efficient initial screening process. In this study, we developed a strategy for the isolation of polyphosphate-accumulating organisms (PAOs) by combining (i) nontoxic fluorescence staining of polyphosphate granules in viable microbial cells and (ii) fluorescence-activated cell sorting (FACS) for the rapid detection and collection of target cells. To implement this screening approach, cells from wastewater sludge samples were stained with 4’6-diamidino-2-phenylindole (DAPI) to target cells with high polyphosphate (polyP) accumulation. We found a staining procedure (10 μg/ml of DAPI for 30 min) that can visualize polyP granules while maintaining viability for the majority of the cells (>60%). The polyP positive cells were recovered by FACS, purified by colony isolation and phylogenetically identified by 16S rRNA gene sequencing. Follow-up analysis confirmed that these isolates accumulate polyP, indicating that DAPI can be implemented in staining living cells and FACS can effectively and rapidly screen and isolate individual cells from a complex microbial community.

## Introduction

The genetic and metabolic diversity of microbial species provide a wealth of possibilities for biotechnological applications. Currently, highly effective isolation methods are largely based on selection by growth capabilities. For example, hydrocarbon-degrading bacteria can be enriched and isolated by using selection media with hydrocarbons as a sole carbon source (Head et al., 2006; Yakimov et al., 2007). In contrast, selectively isolating microorganisms that accumulate desirable intracellular compounds, such as high-valued organic chemicals or environmental pollutants (such as heavy metals), can be challenging, as these organisms may not grow rapidly or may not grow in a unique environment that can be utilized as a selection parameter or selective pressure.

Advances in omics approaches such as single-cell genomics have resulted in the identification of industrially interesting bacteria (Gawad et al., 2016; Rinke, 2018). However, the isolation of microorganisms that accumulate valuable compounds is often a result of laborious individual strain screening, hypothesis-driven isolation methods, or by chance discovery. Persistent screenings of bacterial isolates from seaweed led to the identification of a strain capable of polyhydroxyalkanoates (PHA) production from starch, after investigation of numerous strains for PHA accumulation (Han et al., 2014). Identification of a dominant phylotype *Dechloromonas* in a wastewater treatment plant using 16S rRNA sequencing followed by screening for bacterial strains allowed the isolation of polyphosphate-accumulating strains (Terashima et al., 2016). In one study, enrichment of butanol-tolerant strains led to the identification of a bacterium in the family *Erysipelotrichaceae* that accumulates large amounts of long-chain free fatty acids, with capabilities of retaining these acids under starvation conditions (Katayama et al., 2014). In order to avoid laborious screening of single strains during targeted isolation endeavors, establishing a method to initially enrich for strains showing promising characteristics directly from a mixed population would vastly accelerate the downstream screening process. However, methods to analyze accumulation of specific compounds in individual living cells from a community, followed by recovery and isolation, are still limited.

In this study, we propose a strategy for the isolation of polyphosphate-accumulating organisms (PAOs) by coupling (i) specific staining of an intracellular accumulate without compromising cell viability, and (ii) rapid detection and sorting of target cells by using fluorescence-activated cell sorting (FACS), for which wastewater sludge samples were utilized. During wastewater processing, the removal of phosphate is enhanced by taking advantage of some microorganisms that uptake more phosphate than necessary for growth, leading to the accumulation of polyphosphates (polyP) as an intracellular storage compound (Mino et al., 1998). The use of PAOs in wastewater treatment is a sustainable and economical process and provides a renewable source of phosphate (Yuan et al., 2012). However, many knowledge of PAOs are based on microbial community analyses and not on single strains (Lanham et al., 2018; Nielsen et al., 2019). Although *in situ* data is crucial in identifying and characterizing PAOs, isolating and culturing PAOs will allow for more in-depth experiments. For this reason, our approach focuses on the isolation and cultivation of candidate PAOs from a wastewater sample using fluorescent staining and FACS. The stain 4’6-diamidino-2-phenylindole (DAPI) is frequently used to visualize cellular polyP granules, as it emits a green-yellow fluorescence, which is distinct from the blue fluorescence emitted from DAPI-stained DNA (Tijssen et al., 1982; Streichan et al., 1990; Aschar-Sobbi et al., 2008; Omelon et al., 2009; Schlagenhauf et al., 2016; Correa Deza et al., 2017; Voronkov and Sinetova, 2019). DAPI-staining coupled with fluorescent in situ hybridization followed by microscopy and flow cytometry have been frequently used for fingerprinting and identification of PAOs in various environments (Kong et al., 2005; Günther et al., 2009). However, there have been no reports for the application of DAPI-staining for the isolation of PAOs, since DAPI-staining, which is often accompanied by cell fixation, has been considered to compromise cell viability.

FACS enables rapid screens, with capabilities of analyzing thousands of cells per second. For example, FACS has been utilized for the characterization of microbial community structures and the assessment of live/dead cells from environmental samples (Emerson et al., 2017; Van Nevel et al., 2017; Perera et al., 2019). FACS has also been used in combination with heterologously expressed fluorescent proteins as markers to screen for NADPH-dependent enzymes, phenol-degrading enzymes, amino acid production enzymes and metabolite-responsive transcriptional regulatory proteins (Uchiyama et al., 2005; Uchiyama and Miyazaki, 2013; van Rossum et al., 2013; Eggeling et al., 2015; Mahr et al., 2015; Meier et al., 2016; Bott and Eggeling, 2017; Emerson et al., 2017; Van Nevel et al., 2017; Frei et al., 2018; Ngara and Zhang, 2018; Perera et al., 2019). However, there are only a few studies that utilize FACS for isolation of novel microorganisms from environments. Zengler et al. (2002) achieved isolating viable strains by encapsulating single cells in gel microdroplets and collecting those showing growth by microcolony formation using flow cytometry. Kalyuzhnaya et al. also sorted metabolically active cells using a redox-sensing probe, targeting and successfully isolating methanotrophs (Kalyuzhnaya et al., 2008). However, the targets in these studies were simply active microorganisms and there are currently no reports of application of FACS for the isolation of microorganisms that accumulate a valuable intracellular product, including polyP.

In this study, we developed a rapid method using DAPI staining and FACS for the isolation of PAOs from a complex microbial community. We examined the viability of the cells after DAPI staining and determined a condition that allows for the majority of stained cells remain alive, enabling effective isolation of PAOs after FACS-based phenotype screening.

## Materials and Methods

### Sample Collection and Culture Growth Conditions

The sludge sample utilized for flow cytometry was collected from the oxidation ditch wastewater treatment plant (WWTP) in Okishima, Omihachiman, Shiga prefecture, Japan during the aerobic phase (Terashima et al., 2016). In short, the sludge receives domestic wastewater and is operated with a repetitive cycle of 4 h aerobic (>0.5 mg L^–1^ dissolved oxygen) and 6 h anaerobic phases (<0.5 mg L^–1^ dissolved oxygen). The annual average of biological oxygen demand in the influent/effluent is 167 ± 93/5.0 ± 1.2 mg L^–1^. The total nitrogen and total phosphorus in the influent/effluent are 28.8 ± 6.2/4.3 ± 1.8 mg L^–1^ and 3.9 ± 1.6/0.6 ± 0.2 mg L^–1^, respectively. The influent wastewater pH was 6.7–7.7 (average 7.1) and the effluent wastewater pH was 7.0–7.6 (average 7.4). The plant is located outside with no temperature controls and sampling was conducted when the water temperature was approximately 20°C. In order to allow sufficient time for phosphate uptake, sludge samples were pre-cultured under aerobic conditions at 25°C for by inoculating 4 ml of sludge samples into 8 ml of acetate medium and cultured for 4 h prior to FACS analyses. Sodium acetate was added to the medium because most PAOs prefer short chain fatty acids as a growth substrate (Shen and Zhou, 2016). For the growth medium, sodium acetate was added to a final concentration of 10 mM to the basal media [pH 7, containing per liter of distilled water: 1 g of NH_4_Cl, 0.1 g of MgCl_2_⋅6H_2_O, 0.08 g of CaCl_2_⋅6H_2_O, 0.6 g of NaCl, 2.72 g KH_2_PO_4_, 7.16 g Na_2_HPO_4_⋅12H_2_O, 0.1 g of yeast extract, and 10 ml each of vitamin solution and trace metal solution (Kato et al., 2016)].

### Microscopic Observation of Intracellular PolyP Using DAPI Stain

PolyP and DNA were visualized using fluorescent dye DAPI. 1 ml of sludge samples or individual strains were collected by centrifugation (8,000 g, 5 min) and resuspended in 1 ml of phosphate-buffered saline (PBS) solution and stained for 30 min with DAPI (10 μg/ml final concentration). Cells were imaged using a DMI 4000B Leica fluorescence microscope with a 340–380 nm excitation filter and 425 LP emission filter. Both polyP and lipid inclusions are known to emit in the 450–650 nm range when excited at 360 nm, but lipid inclusions can be easily distinguished from polyP, as their fluorescence intensity is much lower and rapidly fades within seconds (Streichan et al., 1990; Serafim et al., 2002; Seviour et al., 2008; Mesquita et al., 2013). Therefore, all images photographed were exposed to excitation light for at least 1 min before imaging in order to detect consistent, long-lasting bright green-yellow fluorescence from polyP.

### Recovery Test After DAPI Staining

For the viability test after DAPI staining, overnight *E. coli* (strain K-12) cultures and the sludge cultures grown for 4 h as well as for 24 h were utilized. Each sample consisted of three biological replicates and cells were grown in acetate-containing medium as described above. DAPI staining was performed in PBS buffer, as described above, and unstained controls were processed in parallel. After staining, serial dilutions of cells were plated out onto acetate-containing agar plates and incubated at 25°C. Recovered colonies were counted (minimum ∼100 colonies per plate) after 2 days for the *E. coli*-containing plates and after 4 days for the sludge-containing plates. Percent recoveries were determined by dividing the colony numbers of stained sample to the numbers from the unstained control.

### FACS Enrichment of PolyP-Containing Cells

After 4 h of growth in acetate media, cells were vortexed (3 times, 1 min) to break apart cell clumps and filtered through a 40°μm sieve and spun at 8,000 g for 5 min. Cells were resuspended in phosphate-buffered saline (PBS) solution and stained with DAPI and immediately processed by a BD FACS Aria II for sorting. For FACS, a forward scatter threshold of 800 was used and DNA was monitored using a 355 nm UV excitation laser and emission filter of 425–475 nm. PolyP fluorescence was monitored using 405 nm violet laser with an emission filter of 585–625 nm. The voltages used for the forward scatter, DNA and polyp lasers were 277, 515, and 588, respectively. Cells were sorted at a rate of approximately 5,000 cells/s. 1,2% of the cells that showed highest polyP fluorescence relative to the DNA fluorescence were collected by drawing a FACS collection gate and named “PolyP” fraction. As a control, all types of cells detected by FACS regardless of polyP fluorescence intensity were also collected as the “all events” fraction. Over half a million cells were collected (1,2 ml was collected at a concentration of 0.5 × 10^6^ cells/ml) for each sample and a fraction of the collected cells were plated out onto acetate medium agar plates for recovery (replicates of suspension containing 1,000 cells, 100 cells, and 10 cells were plated out). After 4 days of incubation at 25°C, colonies were randomly picked and transferred to clean plates for further analysis.

### Identification of Colonies Recovered After FACS by 16S rRNA Gene Sequencing

The picked colonies were identified via colony-PCR and 16S rRNA gene fragment sequencing. PCR amplification was performed with 27F and U533R primers as described previously (Kato et al., 2010, 2016). PCR products were sequenced at the Biomedical Center of TAKARA Bio Inc with the Applied Biosystems 3730xl DNA Analyzer. The ∼500 bp 16S rRNA gene fragment sequence of each colony analyzed were classified to phylotypes with a cut-off value of 97% sequence identity using BLASTClust (Altschul et al., 1997). The phylotypes were phylogenetically classified with the Classifier program in the Ribosomal Database Project (Wang et al., 2007) and were compared to sequences in the GenBank nucleotide sequence database using the BLAST program (Altschul et al., 1997).

### Accession Numbers

The nucleotide sequence data obtained in this study have been submitted to GenBank database under Accession Nos. LC198009–LC198038.

## Results and Discussion

### Sludge Samples Contain PolyP-Accumulating Strains and Cells Remain Viable After PolyP Staining by DAPI

**FIGURE 1:**
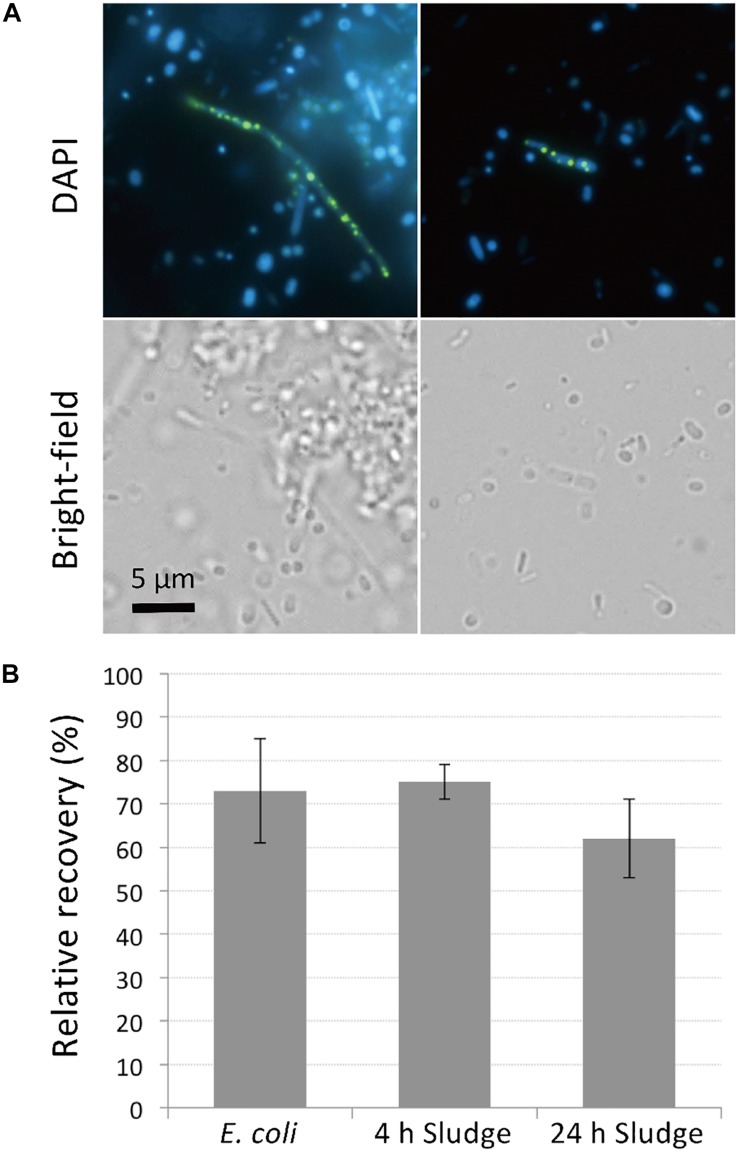
DAPI stains DNA and polyP while maintaining cell viability. **(A)** DAPI-stained images of sludge sample. Top panels show fluorescence images using DAPI filter and bottom panels show bright-field images. DNA emits a blue fluorescence and polyP emits green-yellow fluorescence. **(B)** Most cells recover and generate colonies after DAPI staining. Recovery rates were compared between DAPI-stained and unstained cells by colony counting. Here, the relative recovery rates (% recovery of DAPI-stained cells relative to unstained cells) are shown. Error bars indicate standard deviations of three replicate experiments.

PolyP-accumulation is known to occur under aerobic conditions (Mino et al., 1998). In order to allow for sufficient polyP accumulation prior to DAPI staining, we cultured the sludge samples in phosphate-rich acetate-containing medium under aerobic conditions for 4 h. The presence of polyP-accumulating cells was initially confirmed using microscopy of sludge samples grown overnight and stained with DAPI. DAPI concentration of 1 μg/ml was too low to clearly visualize polyP granules (data not shown), but at a concentration of 10 μg/ml, clear green-yellow fluorescence stemming from polyP granules could be observed (Figure 1A). Additionally, the effect of DAPI staining on cell viability was investigated by plating stained cells on agar plates. Tests using *E. coli* showed that 70% of cells remained viable and formed colonies on plates after staining with 10 μg/ml DAPI for 30 min (Figure 1B). Higher DAPI concentration (100 μg/ml) was also tested in preliminary experiments, which decreased the recovery rate of the cells by approximately half. For the sludge samples, 60–75% of cells retained growth capabilities after 10 μg/ml DAPI staining, showing no significant differences to recovery rates observed for *E. coli* (Figure 1B). These results suggest that DAPI concentration at 10 μg/ml is optimal for polyP detection without compromising cell viability.

### FACS Enrichment and Recovery of PolyP-Accumulating Cells

**FIGURE 2:**
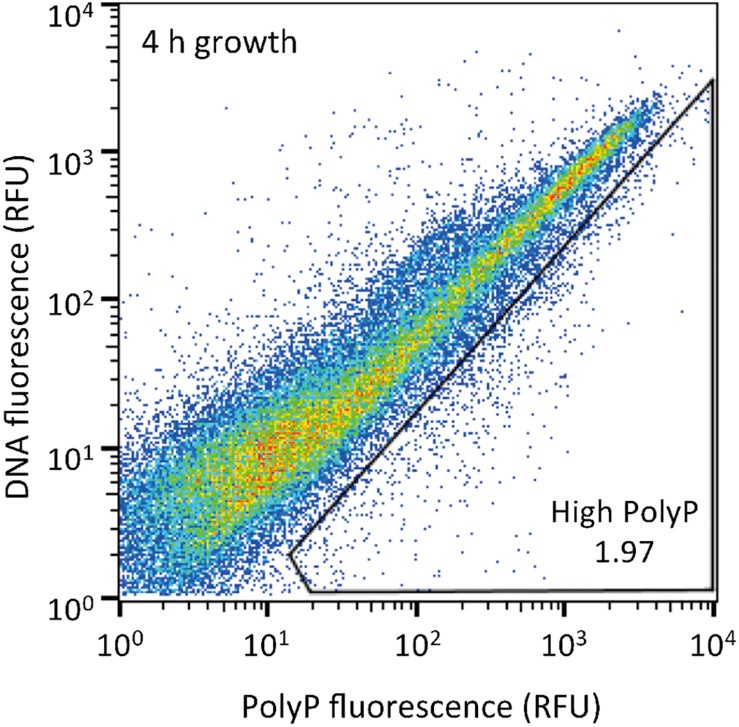
Sludge samples after 4 h of growth in acetate media were stained with DAPI and the top 1,2% of cells with increased green-yellow polyP fluorescence relative to DNA fluorescence were isolated. 10,000 cells are plotted on each graph and the gate drawn onto each plot was used to isolate high polyP cells. The number in the gate represents the % of cells within the gate. RFU, relative fluorescence units.

**FIGURE 3:**
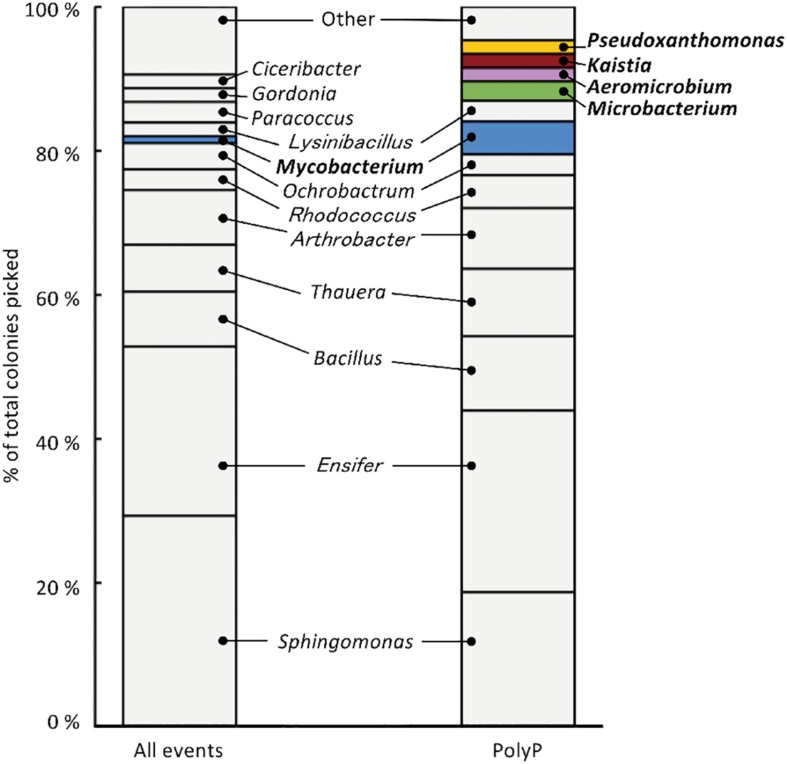
213 isolates were analyzed using 16S rRNA gene sequencing, resulting in the identification of 27 phylotypes (with a cut-off value of >97 %). “All events” indicate control fraction collected by FACS containing all cells and “PolyP” indicates strains collected from the fraction of cells showing high green-yellow fluorescence. Phylotypes that are colored in the graph were three-fold or more enriched in the polyP samples or were exclusively identified in the enrichment fraction by two or more colonies.

The sludge sample was pre-cultured for 4 h, stained with DAPI, and subjected FACS analysis to enrich polyP-accumulating strains. The sludge sample showed a linear distribution between DNA fluorescence and polyP fluorescence (Figure 2). In order to avoid simply collecting large cells based only on high polyP fluorescence signal, cells that showed increased polyP fluorescence relative to the DNA fluorescence were collected. The collection gate was drawn diagonally to encompasses the 1,2% of cells deviating from the linear distribution due to increased polyP fluorescence relative to the DNA signal. These cells in the “polyP” gate were designated as polyP-positive cells and collected, along with collecting all cells (“all events”) as a control, and plated onto acetate medium agar plates. After 2 days, around 30–40% of cells generated colonies on the plates relative to the cells plated out according to numbers obtained from the FACS count. Colonies were identified using colony PCR and 16S rRNA gene fragment sequencing. For the polyP-positive samples, a total of 107 colonies, and for the control samples, 106 colonies were sequenced. Among 213 total colonies analyzed, 27 different phylotypes (with cut off value of >97% identity) were identified (Figure 3 and Supplementary Table S1).

Strains that are candidates for high polyP accumulation were identified by comparing the number of colonies identified for each phylotype between the polyP-positive sample from the “polyP” sort fraction and the “all events” control sample. This approach was taken in order to identify phylotypes that are actually enriched in the “polyP” fraction instead of the distribution tail of a particular phylotype that happened to fall into the sorting gate. Five phylotypes showed a three-fold or more enrichment in the number of colonies in the polyP-positive sample (with a minimum of two isolates), and within these five enriched phylotypes, four were exclusively identified in the polyP-positive samples (Figure 3). These five phylotypes stem from the phyla *Actinobacteria*, *Alphaproteobacteria*, and *Gammaproteobacteria* and are classified into genera *Aeromicrobium* (2 colonies in “PolyP”), *Microbacterium* (3 colonies in “PolyP”), *Mycobacterium* (1 colony in “All” and 5 colonies in “PolyP”), *Kaistia* (2 colonies in “PolyP”), and *Pseudoxanthomonas* (2 colonies in “PolyP”) (Supplementary Table S1). Each of the genus is represented by a single OTU.

### FACS-Enriched Phylotypes Show Consistent PolyP Accumulating Phenotype

**FIGURE 4:**
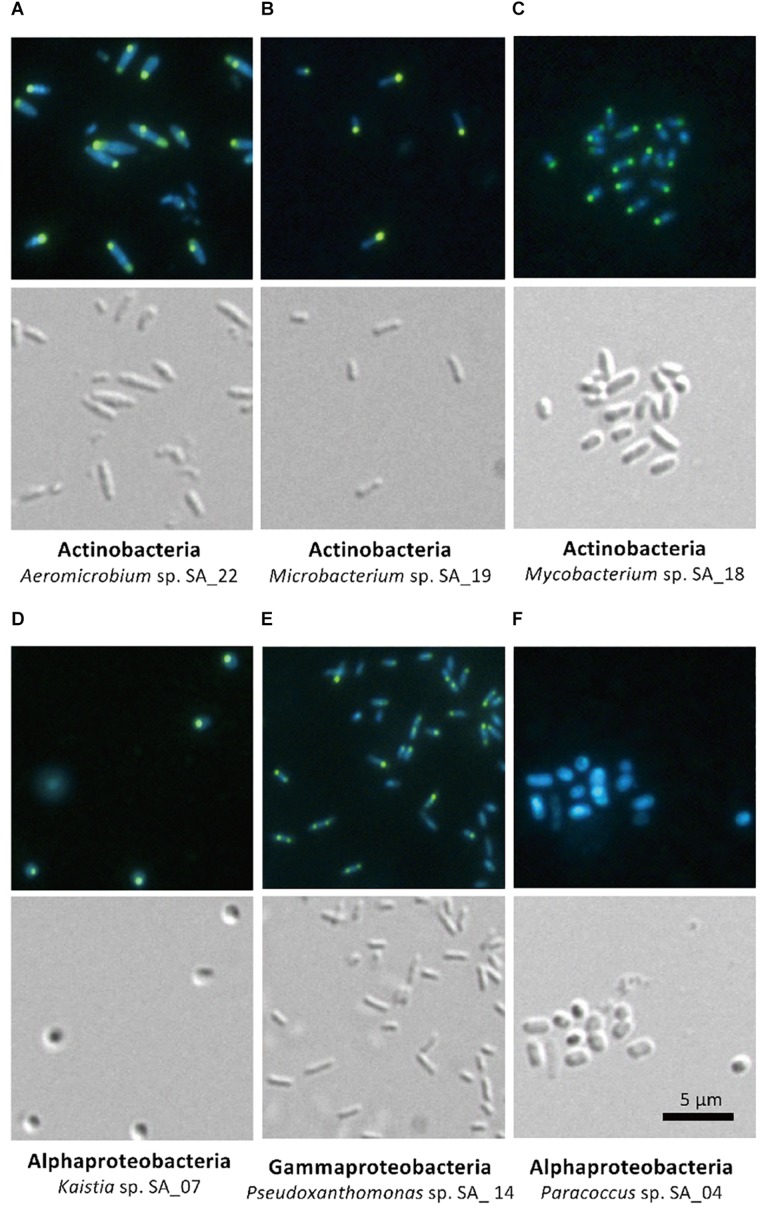
Microscopy of FACS-isolated strains. The top panel for each strain shows DAPI-stained cells and the bottom panel shows differential interference image. Each set of picture is labeled below with the phylum and the strain name. **(A–E)** Strains enriched in the high-polyP sort. **(F)** *Paracoccus* strain, not detected in the high-polyP fraction after 4 h cultivation.

The five isolates (*Aeromicrobium* sp. SA_22, *Microbacterium* sp. SA_19, *Mycobacterium* sp. SA_18, *Kaistia* sp. SA_07 and *Pseudoxanthomonas* sp. SA_14) that were enriched in the polyP-positive population were cultured and re-stained with DAPI and observed using a fluorescence microscope (Figures 4A–E). As a control, we also imaged *Paracoccus* sp. SA_04, which was absent from the colonies recovered from the “polyP” sort, but was present in the “all events” FACS sort of the sludge samples (this strain made up ∼3% of the population in the “all events” FACS control sort, but was 0% in the “polyP” enrichment sort) (Figure 4F). For cells enriched in the “polyP” fraction, microscopy clearly indicated the presence of intracellular granules emitting green-yellow fluorescence (Figure 4A–E). All of these isolates were rod or coccus-shaped and contained one or two large polyphosphate granule(s) in the majority of the cells. On the other hand, the *Paracoccus* sp. SA_04 did not show green-yellow fluorescence (Figure 4F). These results indicate that by isolating the upper 1,2% of high polyP fluorescing cells by FACS, we were able to enrich for isolates capable of polyP accumulation. The enrichment and isolation of polyP-accumulating phylotypes from wastewater microbial community inoculated in acetate-medium contained bacteria in the phyla *Actinobacteria*, and subphyla *Gammaproteobacteria* and *Alphaproteobacteria* (Figure 3). Culture-independent approaches have identified *Betaproteobacteria* related to the group *Rhodocyclus* as being dominant PAOs in many activated sludge systems, as well as members from the *Gammaproteobacteria* and a variety of *Actinobacteria* (Hesselmann et al., 1999; Kong et al., 2005; Seviour et al., 2008; He and McMahon, 2011; Kawakoshi et al., 2012; Nguyen et al., 2012, 2015; Mielczarek et al., 2013; Nielsen et al., 2019). However, it is important to note phylogenic closeness does not necessarily indicate polyP accumulation, as a close relative to a PAO *Candidatus* Accumulibacter phosphatis, a member of the order *Rhodocyclus*, does not accumulate polyP in situ (Albertsen et al., 2016). Members of *Alphaproteobacteria* have been identified in activated sludge systems in many studies, but have not been described to accumulate substantial polyP, (Wong et al., 2004; Beer et al., 2006; Ahn et al., 2007; Zhang et al., 2012; Liao et al., 2013). They may play a role in wastewater phosphate removal, as *Alphaproteobacteria Rhodospirillum rubrum* and *Agrobacterium tumefaciens* are known to accumulate polyP in acidocalcisomes, and members of the *Magnetococcaceae* family also have been found to accumulate high levels of polyP (Seufferheld et al., 2004; Rivas-Lamelo et al., 2017; Kulakovskaya et al., 2019). This, along with our results, further demonstrates the wide phylogenic span of polyP accumulation.

### Concluding Remarks

In this study, we targeted polyP-accumulating bacteria from a wastewater microbial community in order to implement FACS enrichment of living cells as an initial step for phenotype screening of cells accumulating valuable intracellular products. We demonstrated that ∼70% of cells remain viable after DAPI staining, allowing for the phenotype screening and the isolation of polyP-accumulating strains through a single method using FACS. The phylogenies of the isolated colonies were confirmed by 16S rRNA sequencing and colony abundance of each phylotypes was used to identify the PAOs enriched in the polyP-positive fractions. The polyP accumulating capabilities were confirmed by follow-up microscopy. The strains enriched in the polyP sort consistently accumulated large polyP granules. This FACS-based approach allowed for an efficient screening process by identifying promising candidates that led to the successful isolation of PAOs. Further optimization of this method and growth conditions will enable the isolation of a more extensive group of PAOs, including those that were not recovered in our study due to unfavorable growth conditions, sensitivity to DAPI staining or FACS. Comparative genomics on the isolated phylotypes will also further shed light on the polyP metabolism of the isolates. For future endeavors, this approach can not only be adapted to various environmental and wastewater samples for further isolation of PAOs, but also for other phenotype-based screens, such as using non-lethal lipophilic stains (ex. Nile Red) to stain for lipid or PHA accumulation (Lee et al., 2013; Terashima et al., 2015). Furthermore, this approach is not limited to environmental samples, but can be utilized to screen a variety of heterogeneous pool of cells, such mutant isolation after random mutagenesis on a single strain for gene-phenotype linkage analyses.

## Data Availability Statement

The datasets generated for this study can be found in the GenBank database (Accession Nos. LC198009–LC198038).

## Author Contributions

MT, YK, and SK designed the research and analyzed the data. MT conducted the experiments and wrote the manuscript with input from all authors.

## Conflict of Interest

The authors declare that the research was conducted in the absence of any commercial or financial relationships that could be construed as a potential conflict of interest. The handling editor declared a shared affiliation with the authors.
