# Supplementary material for: Rapid Enrichment and Isolation of Polyphosphate-Accumulating Organisms Through 4’6-Diamidino-2-Phenylindole (DAPI) Staining With Fluorescence-Activated Cell Sorting (FACS)
Source: Front Microbiol. 2020 Apr 30;11:793. doi: 10.3389/fmicb.2020.00793 (PMC7203516; doi:10.3389/fmicb.2020.00793)
Supplement: Supplementary file 1 [file Data_Sheet_1.PDF]

Supplemental Table S1. The phylotypes of isolated strains.

Phylo-type	Phylogenetic group		Closest relative (identity, %)	Number of isolates	
	Phylum/Class	Genus		All	PolyP
SA-01	*Alphaproteobacteria*	*Sphingomonas*	AM159534 *Sphingomonas* sp. MN57.2a (98.6)	31	20
SA-02		*Ensifer*	KU877657 *Ensifer adhaerens* strain WJB105 (99.5)	25	27
SA-03		*Ochrobactrum*	KM975672 *Ochrobactrum* sp. WH-13 (99.5)	4	3
SA-04		*Paracoccus*	KJ451483 *Paracoccus* sp. HDM-25 (99.5)	3	
SA-05		*Rhizobium*	JQ659582 *Rhizobium daejeonense* (99.5)	1	1
SA-06		*Ciceribacter*	EU697957 *Rhizobium* sp. CCNWNX0062 (97.8)	2	
**SA-07**		***Kaistia***	**FJ719344 *Kaistia* sp. T94 (99.8)**		**2**
SA-08	*Betaproteobacteria*	*Thauera*	HG513119 *Thauera* sp. 6NLG (98.3)	7	10
SA-09		*Ottowia*	KF751647 *Diaphorobacter* sp. C50 (98.0)	1	1
SA-10		*Variovorax*	JQ689183 *Variovorax paradoxus* (100)	1	1
SA-11		*Cupriavidus*	JX849059 *Cupriavidus basilensis* (100)	1	
SA-12		*Hydrogenophaga*	KX290851 *Hydrogenophaga intermedia* QY7-2 (100)	1	
SA-13		*Achromobacter*	KC577545 *Achromobacter* sp. SR4 (100)	1	
**SA-14**		***Pseudoxanthomonas***	**KF826885 *Pseudoxanthomonas* sp. B14 (100)**		**2**
SA-15		*Pseudomonas*	JQ977107 *Pseudomonas* sp. Cra22 (99.8)	1	

Table S1. Continued.

Phylo-type	Phylogenetic group		Closest relative (identity, %)	Number of clones	
	Phylum/Class	Genus		All	PolyP
SA-16	*Actinobacteria*	*Arthrobacter*	JF700391 *Arthrobacter nicotinovorans* (100)	8	9
SA-17		*Rhodococcus*	KF312643 *Rhodococcus hoagii* (100)	3	5
**SA-18**		***Mycobacterium***	**EU496548 *Mycobacterium* sp. 18 GUW (99.8)**	**1**	**5**
**SA-19**		***Microbacterium***	**HM352852 *Microbacterium hominis* (99.8)**		**3**
SA-20		*Gordonia*	X81927 *Gordonia sputi* (100)	1	1
SA-21		*Gordonia*	JQ658422 *Gordonia* sp. HEXBA02 (99.8)	2	
**SA-22**		***Aeromicrobium***	**Z78209 *Aeromicrobium fastidiosum* (98.4)**		**2**
SA-23		*Streptomyces*	KF881287 *Streptomyces* sp. SCC36 (100)	1	
SA-24		*Rhodococcus*	JQ782925 *Rhodococcus* sp. 7-181 (93.2)	1	
SA-25		*Streptomyces*	KJ742904 *Streptomyces* sp. Sn-23 (99.8)		1
SA-26	*Firmicutes*	*Lysinibacillus*	LK391645 *Lysinibacillus xylanilyticus* (99.8)	2	3
SA-27		*Bacillus*	LC155964 *Bacillus subtilis* strain 6R3-15 (100)	8	11
			Total	106	107
